# Effectiveness of a Food Education Program for healthcare workers: a pilot study in a Total Worker Health© approach

**DOI:** 10.3389/fpubh.2025.1523131

**Published:** 2025-03-12

**Authors:** Reparata Rosa Di Prinzio, Alessia Dosi, Gabriele Arnesano, Maria Eugenia Vacca, Giuseppe Melcore, Mariarita Maimone, Maria Rosaria Vinci, Vincenzo Camisa, Annapaola Santoro, Federica De Falco, Federica De Maio, Guendalina Dalmasso, Eugenio Di Brino, Valerio Pieri, Salvatore Zaffina

**Affiliations:** ^1^Occupational Medicine Unit, Bambino Gesù Children's Hospital Istituti di Ricovero e Cura a Carattere Scientifico (IRCCS), Rome, Italy; ^2^Alta Scuola di Economia e Management dei Sistemi Sanitari (ALTEMS), Università Cattolica del Sacro Cuore, Rome, Italy; ^3^Postgraduate School of Occupational Health, Università Cattolica del Sacro Cuore, Rome, Italy; ^4^Health Directorate, Bambino Gesù Children's Hospital Istituti di Ricovero e Cura a Carattere Scientifico (IRCCS), Rome, Italy; ^5^Department of Business Economics, Università degli Studi Roma Tre, Rome, Italy

**Keywords:** obesity, health promotion, wellbeing, cardiovascular risk, psychological aspects, Total Worker Health approach, health workers, Food Education Program

## Abstract

**Introduction:**

Obesity has been identified as a crucial cause of non-communicable diseases, especially for healthcare workers who often take a brief lunch break with high energy and micro- and macronutrients deficient food.

**Methods:**

Our study aims to investigate the clinical and economic effectiveness of the “Food Education Program” (FEP) among healthcare workers having weight problems. Four questionnaires were administered before and after FEP to explore the risk of psychological injury (“Psychological Injury Risk Indicator”), mental and general health status (“Goldberg's General Health Questionnaire-12” and “Short Form-36 health survey”) and eating behavior (“Eating Attitudes Test”). The Return on Investment (ROI) was calculated on the base of absenteeism reduction in the 1-year period after FEP.

**Results:**

Fifty-one participants (78.4% females, mean age: 52.04 ± 8.94) were included in the study. They were mainly nurses (56.9%). 54.9% were obese and 43.1% overweight. The success rate was 32.1%; the reduction in BMI was more evident in the overweight participants than the obese subjects. A significant reduction of waist-to-hip ratio, glycosylated hemoglobin, total and LDL cholesterol, and an increase in vitamin D was observed (*p*-value: 0.047, 0.002, <0.001, 0.001, and 0.03). Scores on general health significantly improved (*p*-value <0.001 and 0.011). A mean per capita reduction of 3.70 days was observed in 1-year period after the intervention, with a ROI of 6.97.

**Conclusion:**

Food Education Program represents a successful program to improve psychophysical wellbeing of healthcare workers through healthy nutritional plans, also having a notable positive impact on the organization, including its financial accounts.

## 1 Introduction

In the contemporary society, external forces including rapid unplanned urbanization, globalization and population aging may threaten physical and mental health. Seventy-four percentage of all worldwide deaths are attributed to non-communicable diseases (NCDs) which kill 41 million people each year (1). Almost 18 million “premature” deaths occur before the age of 70 years, with cardiovascular diseases being responsible for most of these deaths (1).

Many risk factors have been identified as responsible for NCDs, such as unhealthy diets, physical inactivity, exposure to tobacco smoke, alcohol intake, and air pollution. Chronic NCDs (e.g., high blood pressure, high blood lipid concentrations, increased blood glucose) have been associated to unhealthy lifestyle habits (2). Obesity has been identified as a crucial cause of NCDs, being linked to cardiovascular and cerebrovascular diseases, diabetes, osteoarticular diseases, gastrointestinal diseases, and some neoplasms (3). Notably, “globesity” [the global epidemic of overweight (4)] and “diabesity” [the coexisting status of obesity and diabetes (5)] represent major alarming concerns in Public Health.

Furthermore, psychological distress plays a key role in determining weight problems and incorrect eating styles (6). Emotional eating is a notable example of the complexity of physiological and behavioral interactions between stress and food intake (7). To this extent, poor worklife balance impacts on unhealthy behaviors (e.g., smoking, poor food choices, low levels of exercise, and even reduced sleep time) (8). Moreover, adopting unhealthy lifestyle habits has an impact on organizational performance. It has been estimated that up to 60% of U.S. workers and 45% of European workers are absent from work due to work-related distress (9).

In occupational context, one third of workers have short lunch break, due to increasingly high work rhythms. It leads mostly to unhealthy choices (e.g., fast-consuming foods, refined sugars and saturated fats) which are extremely rich in energy and poor in micro- and macronutrients. The resulting nutritional deficiencies weaken the organism and may open the path for NCDs (10). Moreover, certain occupational risk factors, such as shift work and chronic psycho-physical stress have been associated with a higher frequency of obesity, dyslipidaemia and arterial hypertension, and consequently, of metabolic syndrome (11). In the healthcare sector, shift working has been associated with negative out-comes in dietary patterns. Heavy working schedules often do not allow healthcare workers to follow a balanced lifestyle, being responsible for a negative impact on their weight and eating habits (12).

The workplace is a well-known priority environment to positively influence workers' eating behavior for individual primary prevention (13). Past studies showed that workplace health promotion (WHP) initiatives on healthy lifestyles improve mood, cognitive functions, and overall wellbeing, enhancing concentration and energy levels, reducing stress, improving job satisfaction, thus leading to increased productivity (14–16). Improving nutrition and increasing physical activity has been shown to lessen cardiovascular risk (17). The importance of being well nourished is underlined by International Labor Organization (ILO), which reports that “a diet that is too poor or too rich in the workplace can result in a 20% loss of productivity” (18). Evidence from government bodies clearly confirmed that WHP initiatives and healthy lifestyle programs are an investment for the workforce with a significant impact in terms of reducing sick days and work-related injuries (18).

However, there is a lack of systematic assessment of the interventions in terms of clinic and econometric effectiveness. To fill this gap, our study aims to investigate the clinical and economical effectiveness of an individual educational program focused on an aware food intake among healthcare workers having weight problems. Moreover, we explored gender differences related to the cardiovascular risk profile.

## 2 Materials and methods

### 2.1 The Food Education Program

The “Food Education Program” (FEP) is developed in the hospital in the context of the Workplace Health Promotion plan addressed to the employees from 2021. This plan follows the Total Worker Health^®^ approach, which is defined as a strategy integrating occupational safety and health protection with health promotion to prevent worker injury and illness and to advance health and wellbeing (Table 1) (19).

**Table 1:** Key points of the Food Education Program (FEP).

Team composition	Occupational physician Nutritionist Psychologist
Intervention components	*Dietary plan* Eating behavior. Usual food intake (both liquid and solid) Mealtimes Intolerances and allergies Intestinal activity *Psychological and motivational support* Monthly sessions (individual or joint) Techniques: SMART goals, positive reinforcement, food diary (ABC model)
Phases of FEP	*Enrolment:* Voluntary, based on metabolic parameters *Pre-FEP evaluation:* Initial health assessment *Intervention phase:* 4 months, monthly sessions *Follow-up phase:* s post-intervention evaluation after 2 months
Data collected	*Anthropometric measurements:* Weight, height, BMI, abdominal circumference, WHR *Blood parameters:* Glucose, glycated hemoglobin, insulin, cholesterol, HDL, LDL, ALT, AST, iron, vitamin D, CRP *Mental and physical health status:* Four questionnaires on psychological injury risk, minor psychiatric disorders, general health, and eating attitudes
Assessment	Comparison of variables before and after the intervention

ABC, Antecedent-Behavior-Consequence; ALT, alanine transaminase; AST, aspartate transaminase; BMI, Body Mass Index; CRP, C-reactive protein; FEP, Food Education Programme; HDL, high-density lipoprotein; LDL, low-density lipoprotein; SMART, Specific, Measurable, Achievable, Relevant, and Time-bound; WHR, Waist-hip ratio.

In this scenario, FEP is designed to strike the unhealthy nutritional habits. The FEP is an individual path led by a multidisciplinary team, including the occupational physician, a nutritionist and a psychologist. The intervention is based on a double-faced approach which includes two parts: (i) a diet and (ii) a psychological and motivational support. The psychological support intervention in the Food Education Program (FEP) was structured as a series of four monthly individual or joint sessions involving both the psychologist and the nutritionist. These sessions employed evidence-based strategies to promote behavioral change and adherence to dietary recommendations. The key techniques included SMART goal setting, positive reinforcement, and the use of a food diary within an ABC model framework. Through SMART goal setting, participants were guided to establish goals that were Specific, Measurable, Achievable, Relevant, and Time-bound. This approach was chosen to enhance motivation and increase the likelihood of achieving sustainable health behavior changes (20). Positive reinforcement was employed as a motivational tool to encourage adherence to the dietary plan and to reward progress. This technique leverages principles of operant conditioning, which emphasize the role of rewards in maintaining desired behaviors (21). The use of a food diary helped participants monitor their eating habits, providing valuable insights into meal patterns and emotional triggers. Food diaries have been shown to increase self-awareness and facilitate behavior modification in dietary interventions (22). Specifically, the Antecedent-Behavior-Consequence (ABC) model was applied using food diary entries to analyze eating behaviors. Antecedents (situations or triggers), behaviors (eating patterns), and consequences (behavior outcomes) were identified to better understand and address eating habits (23). These techniques were integrated into the sessions to provide personalized feedback and foster self-regulation, empowering participants to adopt healthier eating habits and sustain long-term changes.

An accurate dietary anamnesis is carried out to collect important data on eating behavior. Usual food intake (both liquid and solid), mealtimes, intolerances and allergies, and intestinal activity are investigated to identify the most suitable Mediterranean pattern of the diet. Moreover, the psychological motivation is essential to keep the path alive. FEP is composed by the following four phases. Firstly, the enrolment of participants, on a voluntary basis, is set up by the occupational physician through a pre-intervention interview, which is useful to ascertain the inclusion criteria and motivational driver of the HCW. The inclusion criteria are represented by altered metabolic parameters. According to the metabolic syndrome diagnosis (24), the following are considered: Body Mass Index (BMI) ≥ 25 (overweight condition); waist circumference > 88 cm in women and >102 cm in men; total cholesterol values > 200 mg/dL and/or LDL values > 160 mg/dL; Triglyceride values > 170 mg/dL; Systolic blood pressure values ≥ 140 mmHg and/or diastolic blood pressure values ≥ 90 mmHg; fasting blood glucose > 110 mg/dL. Exclusion criterion is participants who decided not to follow the program in any of the phases. Subsequently, the pre-FEP evaluation phase is carried out by the whole working group, aimed to obtain an initial assessment of the physical and mental health status. The intervention phase lasts 4 months, with a monthly monitoring by the nutritionist and the psychologist together. The final follow-up phase is performed 2 months after the end of the course with a post-FEP clinical evaluation.

To promote FEP's success, the canteen's food planning was modified by introducing more balanced and nutritious dishes. Vending machines available in the hospital were added on dried fruits and trail mixes as well as the standard sugar choice for coffee and other beverages was set up on the minimum level and fruit juices were eliminated.

During the pre-FEP evaluation and the post-FEP follow-up phases, three categories of data are collected, regarding:

- Anthropometric measurements: weight, height, BMI, abdominal circumference, waist-hip ratio (WHR);
- Blood parameters: glucose, glycated hemoglobin, insulin, total cholesterol, high density lipoprotein (HDL), low density lipoprotein (LDL), alanine transaminase (ALT), aspartate transaminase (AST), iron, vitamin D, C-reactive protein (CRP);
- Mental and physical health status, through the use of four questionnaires related to the risk of psychological injury, minor psychiatric disorders, general health, and eating attitudes.

The clinical effectiveness of the intervention is assessed by comparing the variables before and after.

### 2.2 Study design and setting

A prospective pilot study was conducted in the Occupational Medicine Unit of the hospital between January 2021 and December 2023. Participants were recruited voluntarily during routine check-ups with the occupational physician. Recruitment was based on the identification of alterations in anthropometric measurements and blood parameters. A range of standard demographic and occupational variables were considered to comprehensively analyse the interaction between health outcomes and the work environment. Demographic variables included age and sex, whereas the professional category was used to classify employees in three categories (e.g., nurses, physicians, and technicians). Participants were classified according to the BMI as obese, overweight, and normal weight (25). Modifications of BMI and WHR were recorded and compared among the three groups.

#### 2.2.1 Hematological and lifestyle-related parameters

Hematological parameters included: (i) the lipid profile parameters (e.g., HDL, LDL, and triglyceride levels), (ii) the glucose metabolism (e.g., glycemia, glycosylated hemoglobin, and insulin levels); (iii) indicators of inflammation (e.g., CRP); (iv) iron levels; (v) liver function enzymes AST and ALT; (vi) vitamin D status, categorized into severe deficiency, deficiency, insufficiency, and desirable concentration.

Furthermore, cardiovascular risk factors were examined, including systolic and diastolic blood pressure, WHR and total cholesterol levels. Considering four categories of the risk level (e.g., no risk, low risk, medium risk, and high risk) (26), subjects labeled as high cardiovascular risk were identified and their frequency was compared before and after the program.

The smoking habit was also registered as the main lifestyle-related parameter associated to cardiovascular outcomes.

#### 2.2.2 Questionnaires

During the evaluation meetings at the beginning and at the end of the FEP, four questionnaires were administered.

The 26-item survey called the “Psychological Injury Risk Indicator” (PIRI) investigates psychological injuries related to the workplace and mental health (27). Every question has a Likert (0–6) point rating system. The Italian version was used (28).

The “Goldberg's General Health Questionnaire-12” (GHQ-12) is a 12-item self-administered screening tool used to detect minor psychiatric disorders for the general population (29). GHQ-12 assesses the current mental state and asks whether that differs from the usual state. The questionnaire focuses on both lack of ability to carry out normal functions and appearance of new distressing phenomena. Each question is ranged on a four-point Likert scale and refers to the last 2-week period. The total score can range from 0 to 36 points. Higher scores indicate greater impairment.

The “Short Form-36 health survey” (SF-36) is a 36-item self-completed investigation of general health (30, 31). Each question is ranged on a five-point Likert scale. SF-36 investigates physical health, general health perception, and psychological–emotional health, and contains eight subscales (domains), each scored from 0 to 100 as a weighted sum of the correspondent questions. The higher the score, the better the perceived level of health.

The “Eating Attitudes Test” (EAT-26) is a reliable tool for assessing eating attitudes (32). Scores of 20 or more indicated disordered eating (33).

#### 2.2.3 Absenteeism

Absenteeism from work 1 year before and 1 year after the implementation of FEP was calculated in the group of participants (cases). A group of controls of subjects who did not participate to FEP was similarly evaluated, with each FEP subject being matched to two controls (2n). Case-control matching was set up using age (±1 year), sex and professional category. Sickness absence days (SADs) in the 1-year period were registered and analyzed to assess any changes or trends associated with the intervention.

### 2.3 Statistical analyses

Demographics and occupational variables were described in terms of mean and standard deviation for continuous variables and absolute and relative frequency for categorical variables. After found out that some of the hematological and questionnaire related data were not normally distributed through the Shapiro-Wilk normality test, the pre-post comparison was set up using non-parametric tests, assuming two tailed *p*-value < 0.05 as significant. Statistical analyses were performed using IBM Statistics Package for Social Sciences (SPSS) (version 26.0).

### 2.4 Econometric analyses

The econometric analysis was set up using SAD reduction in the 1-year period as the profit in the Return on Investment (ROI) formula (34). The invested capital was referred to the working time of the multidisciplinary team which carried out the FEP.

## 3 Results

Fifty-one participants over 60 HCWs who were approached agreed to participate and were included in the study; 9 subjects (15.0%) dropped out from the study. They were mainly females (78.4%) and the mean age was 52.04 ± 8.94. The most representative professional category was nurses (56.9%) followed by physicians and technicians (15.7%). They were mostly obese (54.9%) and overweight (43.1), with only one subject having normal weight.

The pre-post comparison showed a success rate of 32.1% for HCWs suffering from obesity (*n* = 9), who gained a normal BMI after the FEP. The success rate founded by the program seems to be good in comparison with similar programs (35). Conversely, although overweight participants did not achieve a BMI within the normal range, they demonstrated a greater reduction in BMI compared to the obese group. However, the obese group exhibited a more pronounced change in WHR. These findings suggest that WHR might serve as a more sensitive indicator of clinical success than BMI when evaluating the program's outcomes (Table 2). In fact, researches indicate that WHR is significantly associated with various health risks, including hypertension, diabetes, and cancer, often outperforming BMI in predictive accuracy. This reliability stems from WHR's focus on fat distribution rather than overall body mass, which can be misleading (36).

**Table 2:** Comparison between obese and overweight subjects.

	**BMI (pre vs. post; pre-post difference)**	***p*-value**	**WHR (pre vs. post; pre-post difference)**	***p*-value**
Obese	33.97 ± 3.19 vs. 32.8 ± 9.48; – (2.32 ± 11.0)	0.538	0.88 ± 0.09 vs. 0.87 ± 0.03; – (0.02 ± 0.04)	0.011
Overweight	27.56 ± 1.72 vs. 5.89 ± 2.14; – (2.85 ± 5.47)	< 0.001	0.83 ± 0.10 vs. 0.82 ± 0.10; – (0.01 ± 0.03)	< 0.001
Normal weight	20.90 vs. 20.10; – (0.80)	-	0.71 vs. 0.73; + (0.02)	-

BMI, body mass index; WHR, waist-hip ratio.

Regarding hematological parameters, the pre-post comparison evidenced a substantial difference in the concentrations of glycosylated hemoglobin, which had a 1.9-point mean reduction, and vitamin D, which registered a 5.8-points increase on average (*p*-values: 0.047 and 0.002, respectively).

Among cardiovascular risk factors, WHR, total cholesterol and LDL showed a significant reduction (*p*-value: < 0.001, 0.001, 0.03). According to literature (26), subjects at high cardiovascular risk moved from 15 to 10 HCWs, with five subjects recovered after the FEP. Moreover, six HCWs have stopped smoking.

Concerning the individual perception of mental and general health, both GHQ-12 and SF-36 scores significantly improved (*p*-value < 0.001 and 0.011, respectively). In detail, physical activity, general health and emotional role limitations significantly improved. Conversely, no significant difference was shown for the EAT-26 score before and after the FEP (Table 3).

**Table 3:** Pre-post comparison of hematological and lifestyle-related parameters and questionnaire scores before and after the Food Education Program.

**Variable**	**Frequency *n* (%) (pre vs. post)**	**Mean ±SD (pre vs. post)**	***p-*value**
**A) Success rate**			
BMI (obese – over-weight – normal weight)	28 (54.9) – 22 (43.1) – 1 (2.0) vs. 19 (37.3) – 22 (43.1) – 10 (19.6)	-	< 0.011
**B) Hematological parameters**			
HDL (mg/dL)	-	57.0 ± 13.3 vs. 55.8 ± 14.0	
LDL (mg/dL)	-	128.5 ± 36.3 vs.	0.793
Triglyceride (mg/dL)	-	123.8 ± 33.9	0.03
Glycosylated hemoglobin (mg/dL)	-	112.7 ± 108.2 vs.	0.628
Glycemia (mg/dL)	-	118.6 ± 96.6	0.047
Insulin μU/mL	-	38.0 ± 8.5 vs. 36.1 ± 3.7	0.886
CRP (mg/L)	-	96.9 ± 8.5 vs. 93.4 ± 12.0	0.201
Iron (μg/dL)	-	13.2 ± 7.8 vs. 12.9 ± 8.7	0.511
AST (U/L)	-	0.5 ± 0.9 vs. 0.6 ± 1.3	0.841
ALT (U/L)	-	80.9 ± 34.9 vs. 79.1 ± 28.3	0.692
Vitamin D (ng/mL) (severe deficiency – deficiency – insufficiency – desirable concentration)^a^	1 (2.2) – 14 (31.1) – 16 (35.6) – 14 (31.1) vs. 0 (0.0) – 2 (5.3) – 17 (44.7) – 19 (50.0)	21.4 ± 9.4 vs. 20.4 ± 8.3 22.7 ± 14.6 vs. 22.3 ± 16.4 25.8 ± 10.5 vs. 31.6 ± 9.5	0.718 0.002; < 0.001
**C) Cardiovascular risk factors**			
Systolic blood pressure (mmHg)	-	121.7 ± 11.45 vs.	0.199
Diastolic blood pressure (mmHg)	-	125.6 ± 14.0	
WHR	27 (52.9) vs. 24 (47.1)^b^	79.3 ± 6.8 vs. 80.4 ± 10.1	0.817; 0.614
Total cholesterol (mg/dL) (no risk – low risk – medium risk – high risk) High cardiovascular risk	8 (15.7) – 16 (31.4) – 20 (39.2) – 7 (13.7) vs. 13 (25.5) – 20 (39.2) – 14 (27.5) – 4 (7.8) 15 (29.4) vs. 10 (19.6)	0.86 ± 0.10 vs. 0.84 ± 1.0 -	< 0.001; < 0.001 0.001 0.116
Smoking habit	8 (33.3) vs. 2 (11.8)	-	< 0.001
**D) Questionnaire**			
PIRI, total score	-	44.8 ± 23.7 vs. 48.4 ± 28.4	0.488
GHQ-12	-	12.8 ± 6.6 vs. 8.8 ± 5.1	< 0.001
SF-36—total score	-	553.5 ± 136.6 vs.	0.011
Subscale 1—Physical activity	-	595.9 ± 132.6	< 0.001
Subscale 2—Physical role limitations	-	75.4 ± 20.3 vs. 85.1 ± 15.3	0.055
Subscale 3—Physical pain	-	74.0 ± 29.6 vs. 81.3 ± 28.1	0.686
Subscale 4—General health	-	74.5 ± 27.1 vs. 76.8 ± 22.4	0.050
Subscale 5—Vitality	-	59.3 ± 20.1 vs. 63.7 ± 19.8	0.453
Subscale 6—Social activities	-	60.1 ± 18.0 vs. 62.1 ± 19.5	0.415
Subscale 7—Emotional role limitations	-	67.6 ± 21.9 vs. 70.6 ± 25.0	0.001
Subscale 8—Mental health	-	77.2 ± 33.1 vs. 88.6 ± 27.7	0.082
EAT	-	65.4 ± 16.6 vs. 69.7 ± 19.4 9.1 ± 6.1 vs. 8.3 ± 5.2	0.291

ALT, alanine transaminase; AST, aspartate transaminase; BMI, Body Mass Index; CRP, C-reactive protein; EAT, Eating Attitudes Test; GHQ-12, General Health Questionnaire-12; HDL, high-density lipoprotein; LDL, low-density lipoprotein; SF-36, Short Form-36; WHR, Waist-hip ratio.

^a^Severe deficiency: < 10 ng/mL—deficiency: 10–20 ng/mL—Insufficiency: 20–30 ng/mL—desirable concentration: 30–50 ng/mL.

^b^High WHR.

Our findings highlighted gender differences regarding arterial blood pressure, which was significantly higher in males than females both before and after the FEP. Conversely, women had higher blood lipid levels than men and reported more problems in terms of eating attitudes (Table 4).

**Table 4:** Comparison of males and females in terms of hematological and lifestyle-related parameters and questionnaire scores before and after the Food Education Program (only statistically significant parameters are listed).

	**Male**	**Female**	**Mann-Whitney's *U***	***p*-value**
SAP, post	147 ± 11	123 ± 12	64,000	0.012
DAP, post	93 ± 8	79 ± 9	61,000	0.032
Total cholesterol, pre	164 ± 37	209 ± 40	91,000	0.003
Total cholesterol, post	168 ± 37	201 ± 38	106,000	0.032
HDL, pre	48 ± 7	60 ± 13	97,500	0.005
HDL, post	48 ± 10	58 ± 14	98,500	0.018
LDL, post	106 ± 33	130 ± 33	104,000	0.036
EAT score, pre	5 ± 3	10 ± 6	97,500	0.005

DAP, diastolic arterial pressure; EAT, Eating Attitude Test; HDL, high-density lipoprotein; LDL, low-density lipoprotein; SAP, systolic arterial pressure; WHR, waist-hip ratio.

Setting aside the well-known benefits of achieving a normal body weight at the individual level, in terms of direct, indirect, and intangible costs (e.g., quality of life and life expectancy), it is important to emphasize that corporate programs such as the one described in this study also yield significant benefits for employers. These benefits include reductions in both direct and indirect costs (e.g., decreased absenteeism due to fewer requests for medical leave and fewer days off work), as well as reduced presenteeism and a recovery in productivity, particularly in non-sedentary roles that require constant movement, which is especially relevant in healthcare professions.

Our analysis focused exclusively on the direct savings resulting from the reduction in SADs. In the year following the intervention, participants in the intervention group experienced an average per capita reduction of 3.70 sick days compared to the previous year, while the control group showed an average increase of 4.43 days per person. By comparing the per capita value of the reduction in SAD (628.26 euros) with the project cost per individual (78.79 euros), we calculated a return on investment (ROI) of 6.97 (Table 5).

**Table 5:** Sickness absence days and return on investment calculation.

**SADs**	**One-year before (total days)**	**One-year after (total days)**	**Post-pre difference (per capita days)**
Case	258	184	3.4
Control	651	828	11.5
**Invested capital**	**Number of hours**	**Hourly cost**	**Total cost**
Physician	0.5	21.23 €	10.61 €
Nutritionist	6	5.68 €	34.09 €
Psychologist	6	5.68 €	34.09 €
Entire team			78.79 €
**Gross profit**			628.26 €
**Net profit**			549.47 €
**ROI**			**6.97**

ROI, return on investment. Gross profit and net profit are self-explanatory.

## 4 Discussion

Our findings indicate that the Food Education Program is more effective for obese individuals compared to those who are overweight. Research shows that obese individuals tend to lose weight more rapidly than overweight people and the greater the initial weight loss, the greater the rates of weight maintenance over time (37). Faster rates of weight loss can lead to a greater loss of fat-free mass and a smaller loss of fat mass during the dynamic phase of weight reduction (38). Studies have demonstrated that the rate of weight loss is positively correlated with factors such as the individual's age, initial body weight, frequency of dietary counseling, and prescribed energy deficit. Additionally, older and obese individuals who have more frequent contact with counselors and adhere to calories restrictions, tend to lose weight at a faster rate (39).

### 4.1 The improvement of cardiovascular risk profile

In our population the cardiovascular risk profile appears significantly improved after the nutrition intervention, in terms of reduced glycosylated hemoglobin, total and LDL cholesterol and WHR. Acting on reversible cardiovascular risk factors, Food Education Programs have been shown to actively prevent chronic NCDs (40).

According to literature, a proper diet should take into account both the energy and nutrient properties of the food, the type of work performed (sedentary, varied, light, heavy), the working environment (temperature, humidity, etc.), working hours (continuous, shifts), the type of habitual diet (nationality, religion, etc.), and non-work activities (sport, second job, hobbies) (18). For instance, the healthy eating guidelines established that meals eaten while working should be not too abundant, easily digestible, mainly made up of carbohydrates (bread, pasta, rice), legumes, fruit, and vegetables (foods with a high content of mineral salts) and an adequate water supply. In mental work, which is usually associated with a sedentary lifestyle, nutrition must be particularly careful as it consumes energy it is minimal. To this respect, the Mediterranean diet, been recognized by UNESCO as an intangible heritage of humanity thanks to its wide range of beneficial effects, may be the right choice (41). Studies have reported that implementing Mediterranean dietary patterns generates a significant decrease in body weight, BMI and abdominal circumference of up to 40% (42), of total and LDL cholesterol and the reduction of the WHR ratio (43, 44). This contributes to a 25–45% reduction in the risk of cardiovascular morbidity and mortality (e.g., 25–30% lower incidence of type 2 diabetes and 30–40% lower incidence of metabolic syndrome) and to a 20–30% reduction in cancer mortality (42). The Mediterranean diet is not just a way of eating, but a set of knowledge, social habits, and cultural traditions. In the choice of food to be prescribed, preference should be given to a mainly plant-based diet with a meal distribution throughout the day represented by five meals: three main meals (breakfast, lunch and dinner) and two snacks (mid-morning and mid-afternoon). According to this model, establishing a proper meal routine allows to maintain an adequate hunger-satiety rhythm throughout the day and, consequently, to follow a varied and balanced diet, preventing the risk of obesity and related chronic NCDs (41).

In our population we found a significant increase in vitamin D levels in combination with an improved perception of physical activity. In fact, in accordance with the literature, an increase in plasma vitamin D concentration occurs with both indoor and outdoor physical activity (45).

Among participants who lost weight, about 40% also stopped smoking. This result has amplifying effects on the cardiovascular risk profile, since it contributes to low systemic inflammation, as happens with increased physical activity and weight loss (46).

Furthermore, a gender difference has been outlined in fruit and vegetable consumption, which is higher in women and in olders with a higher level of education (47, 48). In literature, gender difference has been linked to psychological health, since higher consumption of fast, fried or sugary foods in women has been associated to depression (49).

### 4.2 The improvement of health perception

In our population the individual perception of mental and general health (GHQ-12 and SF-36 scores) significantly improved.

Physical activity has been outlined as tightly correlated to nutritional aspects, since diets help weight loss. The combination of a balanced diet and regular physical activity is crucial for effective weight loss, with a better outcome than focusing on diet alone (50). Combining a low-calorie diet with regular physical activity not only facilitates achieving optimal weight management but also improves overall health, including better cardiovascular parameters and metabolic outcomes (50, 51). The broad long-term health benefits of integrating physical activity with dietary interventions reinforce the importance of a holistic approach to weight management (52). The combined approach reduces the likelihood of weight regain after initial weight loss (53). Eating schedules are one of the major protective factors for the improvement of general health. Several studies show that structured and balanced diets significantly enhance greater and more sustainable weight loss outcomes compared to unorganized dietary approaches (51, 52).

Moreover, our results underline that the risk for anxiety and depression traits significantly lowered as well as the perception of emotional role limitations improved. This highlights the important role of psychologists in the TWH system. Psychologists contribute by promoting interventions that reduce workplace stress, enhance mental health, and develop a supportive work environment. Their expertise in behavior change is crucial for designing programs that encourage healthy lifestyles and increase resilience among workers. Moreover, psychologists play a key role in organizational assessments and the implementation of policies that support holistic health approaches. Studies have shown that integrating psychological strategies into TWH initiatives leads to improved worker's outcomes and overall organizational productivity (54).

### 4.3 The role of health policies

Health policy and health promotion policies in workplaces play a crucial role in safeguarding the wellbeing of employees, particularly through initiatives focused on food education to prevent cardiovascular diseases and NCDs (55).

According to our results, different actions could be implemented to counteract the consumption of fast/take-away food and increase the availability of healthy food in the workplace. For instance, in-house vending machines could be revamped to offer healthier options. Some ideas may include freshly washed and packaged fruit, natural fruit juices, low-calories snacks (e.g., whole-grain cereal bars or dried fruit), yogurt and ready-to-eat fresh salads (56). Moreover, reducing the price of healthy products could incentivise workers to make better choices (56). The availability of low-calories or hypolipidic menus in the canteens could be proposed by nutrition experts (57). Information materials near vending machines or in the canteen may encourage healthier food choices and give details about the products on sale (58). Nutrition training courses could be organized to educate employees on proper nutrition. Many communication strategies have been suggested to reach the goal of a better eating community, including brochures, motivational telephone interviews and e-mail messages (59). In the context of health policies, the role of the occupational doctor is essential to the purpose of improving the workforce's health status and healthy lifestyle behavior. During the periodical medical surveillance, informative questionnaires could be useful to collect data on eating behavior. The administration of questionnaires before the medical examination could open the opportunity to give a tailored counseling on the theme. A study showed that sending e-mail messages can positively influence eating styles in the healthcare sector (60).

Furthermore, beyond the traditional organizational health policies, the education and training of healthcare professionals could enhance the process of food literacy in the community. Knowledge and skills related to the relationship between food and people, communities, and the environment and detailed nutrition should be integrated into the university courses (61, 62), and also WHP interventions regarding food education could improve the awareness of the importance of nutrition (63). Experiential education is clinically relevant to equip future health professionals with practical skills by converting awareness of system-level issues into self-reflection, hands-on cooking and eating, and cross-disciplinary engagement (64). Moreover, a gender-focused teaching approach may be crucial for the purposes of wellness promotion and nutrition education courses (65).

Thus, integrated health policies that emphasize food education in the training of healthcare professionals in a lifelong learning in workplaces promote healthier lifestyles and reduce the incidence of chronic diseases, contributing to overall workforce health and productivity.

### 4.4 Strengths and limitations of the study

This study has several strengths that enhance its reliability and relevance. One notable strength lies in its methodology, which employed rigorous sampling techniques and comprehensive data collection methods. Furthermore, the inclusion of detailed statistical analyses facilitated a thorough exploration of the relationships under investigation. The study has some limitations, including the potential for recall bias among participants, particularly regarding self-reported data on lifestyle factors and health outcomes. Moreover, while the sample size was adequate for statistical power, larger samples could provide more precise estimates and further strengthen the study's conclusions. Additionally, the voluntary recruitment process, while potentially introducing selection bias by attracting highly motivated individuals, aligns with the program's focus on those willing to actively participate, thereby ensuring the intervention's feasibility and real-world applicability. Despite these limitations, the strengths of the study could provide valuable contributions to the existing literature on the importance of food education intervention on the workplace offering insights that can inform both policy and practice in occupational health and for whom in charge of health promotion interventions.

## 5 Conclusions

Our study showed the positive impact of the nutrition program with a relevant success rate in the healthcare sector. Clinical improvement of blood parameters and individual perception of the health status after the 6-month path were also reflected on the reduced absenteeism and increased productivity in the workplace. Having a systematic approach to the health status is essential to rise the level of organizational wellbeing from a Total Worker Health^®^ perspective.

## Data Availability

The original contributions presented in the study are included in the article/supplementary material, further inquiries can be directed to the corresponding author.
